# Being Bullied at School: Gratitude as Potential Protective Factor for Suicide Risk in Adolescents

**DOI:** 10.3389/fpsyg.2019.00662

**Published:** 2019-03-26

**Authors:** Lourdes Rey, Cirenia Quintana-Orts, Sergio Mérida-López, Natalio Extremera

**Affiliations:** ^1^Department of Personality, Evaluation and Psychological Treatment, Universidad de Málaga, Málaga, Spain; ^2^Department of Social Psychology, Universidad de Málaga, Málaga, Spain

**Keywords:** bullying victimization, suicidal thoughts and behaviors, depressive symptoms, gratitude, adolescence

## Abstract

Bullying victimization has been recognized as a risk factor for social, physical, and psychological problems in adolescence. One promising resource that seems to protect adolescents from adversity and traumatic events is gratitude. However, no analysis of the specific role of gratitude in bullying context has been performed as yet. Thus, the aim of this research was to explore the associations between bullying victimization, gratitude and suicide risk (i.e., depressive symptoms and suicidal thoughts and behaviors) and gender-based differences. We also investigated whether levels of gratitude moderated the relationship between victimization and suicide risk for girls and boys. A convenience sample of 1,617 adolescents (50.5% girls; *M* age = 14.02) participated in this research. Adolescents completed a paper-and-pencil questionnaire about their bullying victimization, gratitude, depressive symptoms and suicidal thoughts and behaviors. Gratitude was found to be negatively related to victimization and suicide risk. While no gender differences were found in gratitude, it was observed that girls reported higher levels of suicide risk. However, the victimization × gratitude interaction contributed to variance in suicide risk, but only for girls: Those girls who were victims of bullying with high gratitude scores reported lower suicide risk than their counterparts who showed less gratitude. Thus, the findings from this present cross-sectional study suggest that gratitude is related to suicide risk in the context of bullying victimization, especially among adolescent girls. Finally, the theoretical and practical implications of our novel contributions to the understanding of gratitude as a protective factor against consequences of bullying victimization are discussed.

## Introduction

Recent studies have indicated that bullying victimization – i.e., when an adolescent is beaten, insulted, threatened, robbed, excluded, or rumored intentionally and repeatedly by some student or several physically or psychologically strengthen; Ortega-Ruiz et al., 2016 – has considerable amounts of negative consequences for both the physical and mental health of adolescents (Povedano et al., 2015; Stapinski et al., 2015). Consistent with these findings, bullying research has shown that victimized adolescents often report high levels of affective disorders and negative health outcomes such as depression and suicidal behavior and ideation (Özdemir and Stattin, 2011; Fredrick and Demaray, 2018). For example, Holt et al. (2015), in a meta-analysis, found significant associations between bullying victimization and suicidal behavior. Similarly, Stapinski et al. (2015) showed that bullying victimization not only caused short-term effects on mental health, but also had a delayed impact on symptoms of depression. Both distal (e.g., depressive symptoms) and proximal (e.g., suicidal ideation) variables are thought to increase the risk of death by suicide among adolescents (Bonner and Rich, 1987; Sareen, 2011; Chang et al., 2017).

Despite the robust association between bullying victimization, depressive symptoms and suicidal behavior and ideation, a common limitation in this area of research is the scarce attention given to examining the influence of personal resources to ameliorate the potentially negative effects of bullying victimization. The organism-environment interaction theory (Lerner et al., 2006) states that not all persons are equally influenced by the same context, and it is the interaction between person and context that contribute to persons’ social and psychological adjustment. Additionally, research on bullying (Zych et al., 2017; Extremera et al., 2018) has suggested that persons with different positive internal resources will respond differently to stress or negative experiences such as victimization. This variation in response to bullying victimization suggests that there may be different profiles associated with victimization. As well, there appear to be consistent gender differences in the relation between victimization and internalizing problems, with females experiencing higher levels of depression and suicidal ideation in consequence of bullying (Brunstein Klomek et al., 2007; Fredrick and Demaray, 2018). However, results concerning the associations between bullying victimization and gender are mixed and unclear. For example, although most studies have found that bullying victimization is more common for girls than for boys, others have found that boys are generally more victimized than girls, and, even, some research has found no gender differences (Zych et al., 2015).

Several researchers (Zhou et al., 2017; Zych et al., 2017; Quintana-Orts and Rey, 2018) have begun to examine how individual positive factors might ameliorate the negative association of bullying victimization and suicide risk. One such positive characteristic and psychological strength, thus far scarcely studied, is gratitude, or thankfulness. This is commonly defined as a two-step process: first, recognizing that one has obtained a positive outcome; and second, recognizing that there is an external source for this positive outcome (Emmons and McCullough, 2003). Some scholars (e.g., Allen, 2018) categorize three different conceptualizations of gratitude: affective trait (disposition across time and circumstances where gratitude is an extension of an individual’s personality), mood (daily variations in overall gratitude), and emotion (a more transitory feeling of gratitude one may feel after a specific situation such as receiving a gift or a favor). In this study, we focus on trait (or “dispositional”) gratitude.

An increasing number of studies shows that dispositional gratitude seems to have far-ranging positive impacts for children and adolescents. For example, studies have found that more grateful adolescents are happier with their school and report better interpersonal relationships (Froh et al., 2010; Mofidi et al., 2014) and develop more prosocial behaviors (Bono et al., 2017).Furthermore, some scholars suggest that gratitude has a robust relationship with both mental health and psychological adjustment (Wood et al., 2010). For example, several studies have shown that gratitude is linked to lower depressive symptoms (Neto, 2007; Petrocchi and Couyoumdjian, 2016; Disabato et al., 2017) and reduced suicidal ideation and suicide attempts (Li et al., 2012).

Although gratitude is a positive characteristic observed in all individuals, there are studies that have found differences between males and females regarding gratitude, in both adolescents and adults. Such studies (Neto, 2007; Petrocchi and Couyoumdjian, 2016; Disabato et al., 2017; Yost-Dubrow and Dunham, 2018) found that girls and women report higher levels of gratitude than their male counterparts. However, other studies (Freitas et al., 2011; Ruch et al., 2014; Wang et al., 2015) did not find these differences.

Some studies focusing on the importance of personal factors in bullying have found their protective value against the development of internalizing (e.g., depression) and externalizing problems (e.g., suicide). For example, some authors suggest that forgiveness is an emotion-focused coping strategy to help victims alleviate the negative outcomes of being bullied (Freedman, 2018) and have lower levels of depression, anxiety, anger, or hostility (Toussaint et al., 2015). On the same vein other authors highlight other resources such as self-esteem (Turner et al., 2010), resilience (Sapouna and Wolke, 2013) and optimism (Rigby, 2003) as relevant factors that may successfully influence in overcoming victimization experiences. According to Fredrickson’s (1998) broaden-and-build theory, people with positive emotions have broad-ranging thoughts and actions, which allow individuals access to resources and may help them to resist negative life choices and decreasing difficulties. In this regard, gratitude is suggested as another protective factor linked to more positive emotions and appraisals against negative experiences and psychological difficulties (e.g., Disabato et al., 2017; Nezlek et al., 2018). However, gratitude has been neglected in bullying research, and, as a result, it is not currently known how some bullied adolescents manage bullying victimization to recover from its impact and stay healthy over time considering their levels of gratitude. In order to address this gap, the purpose of this research is to explore whether gratitude is a positive resource that could protect against negative outcomes such as other personal resources do.

### The Present Study

To the best of our knowledge, research on the possible protective role of gratitude in the relationship between bullying victimization and mental health in adolescence has been not empirically assessed. For that reason, the aim of this study was to extend our understanding of the links among bullying victimization and suicide risk (depression and suicidal behavior and ideation), in three regards. Our first objective was to explore the relationships between bullying victimization, gratitude and suicide risk in an adolescent sample, with the aim to extend our understanding of bullying victimization experiences in this age group. Our second aim was to explore gender-based differences in the association between bullying victimization and mental health and the relative role of gratitude at promoting psychological adjustment. Thirdly, we endeavored to determine whether there is a significant victimization × gratitude interaction effect in predicting suicide risk.

As aforementioned, gratitude has been shown to be positively linked to adolescents’ psychological adjustment (Froh et al., 2008, 2009; Bono and Froh, 2009). Thus, we expected bullying victimization to be positively related to suicide risk, whereas we predicted gratitude would be negatively related to suicide risk (H1). In addition, regarding gender differences, studies on bullying victimization (Froh et al., 2009; Fredrick and Demaray, 2018) suggest consistent differences between boys and girls in gratitude, depressive symptoms and suicidal behavior and ideation. Therefore, we expected results consistent with these findings (H2), with girls reporting higher levels of suicidal thoughts and behaviors, depression and gratitude compared to boys. Regarding victimization, we explored the results caused by mixed results of research. Finally, gaining more insight into the specific gender pattern between gratitude and suicide risk may help improve our understanding of the manner and methods of conducting anti-bullying prevention and intervention strategies. Thus, we tentatively hypothesized that, independent of gender, adolescents with higher levels of gratitude would report lower levels of suicide risk. That is, we expected to find that gratitude served as a buffer between bullying victimization and suicide risk (depressive symptoms and suicide) in both adolescent males and females (H3).

## Materials and Methods

### Participants

A convenience sample of 1,617 adolescents (50.5% female) from several public high schools in Málaga (Andalusia, Spain) participated in this study. The mean age was 14.02 years (*SD* = 1.46; range 12–17). A 83.4% of the sample was Spanish. Regarding the academic level taught, 29.6% attended classes of the 1st year of Compulsory Secondary Education; 28.1% attended classes of the 2nd year; 22.2% the 3rd year and 12.3% the last course. A 7.7% of the sample attended classes at A level.

### Procedure

Principals of the schools were responsible for reporting and consulting to the parents about the study. Parents were asked to provide an informed consent to use the data anonymously in the present research. A written consent for participants was provided to school authorities, who made the last decision on their participation. Besides, principals provided written informed consent for the conduct of the study. There was no parental refusal for adolescents’ participation. The data was collected in classrooms during a 1-h lesson, always with the presence of one of the researchers and at least one schoolteacher, and with guarantees of the participants’ voluntariness and anonymity. All participants were encouraged to answer honestly. The study was carried out in accordance with the ethical principles for psychological research involving human subjects and was approved by the Research Ethics Committee of University of Málaga (62-2016-H).

#### Bullying Victimization

Bullying victimization was measured using the Victimization subscale of the European Bullying Intervention Project Questionnaire (EBIP-Q; Brighi et al., 2012). The EBIP-Q subscale comprises seven items representing the frequency of bullying over the previous 2 months. All responses were on a 5-point Likert scale ranging from 0 for “never” to 4 for “more than once a week.” The Spanish version was used (Ortega-Ruiz et al., 2016). For this sample, Cronbach’s alpha for the bullying victimization subscale was 0.82.

#### Suicide Risk

To assess suicide risk in adolescents we used two measures concerning suicide and depression. This choice was made for two reasons. Firstly, because of “suicidal tendencies and behaviors are defined as a continuum of behaviors, with suicidal ideation on one end of the continuum, and death by suicide on the other end” (Mazza, 2006; Fredrick and Demaray, 2018) and secondly, due to the solid involvement of depressive symptoms in suicide (Konick and Gutierrez, 2005; Cukrowicz et al., 2011; Cheung et al., 2018; Quintana-Orts and Rey, 2018).

For *depressive symptoms*, we used the Depression Inventory Short Version (CDI-S; Kovacs, 1992). The Spanish version by Del Barrio and Carrasco (2004) was used. CDI-S is a 10-item measure that assesses the severity of depressive symptoms in adolescents. The items are enunciated, in three sentences that represent three levels of intensity of depressive symptomatology. The total scores range from 0 to 20. The instrument has high internal consistency in Spanish samples (de la Vega et al., 2016). Higher scores on the CDI-S indicate greater depressive symptomatology. The internal consistency for the CDI-S in this study was 0.76.

For *suicidal thoughts and behavior*, the Suicidal Behaviors Questionnaire–Revised (SBQ-R; Osman et al., 2001) was used. The SBQ-R has four items and provides an indication of overall suicidality. Participants were asked to respond to different aspects relating to suicide: lifetime suicidal thoughts and suicide behaviors (six levels of answers); frequency of suicidal ideation in the last year (five levels of frequency); suicidal intention and likelihood suicidal attempt in the future (five and six levels of answer, respectively). Higher scores on the SBQ-R indicate greater suicidality. The SBQ-R was validated for Spanish adolescents by Rey et al. (2018a) and was found to have adequate psychometric properties. In the current research, the Spanish version showed good internal consistency (Cronbach’s alpha = 0.87).

#### Gratitude

The Gratitude Questionnaire (GQ; McCullough et al., 2002). The Spanish version by Rey et al. (2018b) was used to assess gratitude. The Spanish version, validated for adolescents, is a five-item self-report scale. The statements were rated on a 7-point Likert-type scale (1 = strongly disagree to 7 = strongly agree; e.g., “I have so much in life to be thankful for”). Higher scores indicate greater levels of gratitude. This scale has adequate psychometric properties. In the present study, the internal consistency of the scale was 0.79.

### Statistical Analyses

Data was analyzed using the program SPSS (version 22). First, descriptive analyses were used to describe the demographic information of the sample. Next, we adopted Pearson’s correlation analyses to examine the associations among research variables in the total sample. Participants’ gender differences in the research variables were examined by using Student’s *t*-test. Finally, to analyze the potential buffering effects of gratitude in both boys and girls, separate moderation analyses were conducted for each group using the process macro (Model 1) developed by Hayes (2013). The significance of the indirect effect at different levels of the moderator was tested using bias-corrected bootstrap confidence intervals (CIs) at 95% (5,000 random samples).

## Results

### Demographic Characteristics

The demographic information of the participants is presented in Table 1.

**Table 1:** Demographic characteristics of the sample.

	Percent	*n*
*Gender*		
Males	49.5	800
Females	50.5	817
*School grade*		
1st compulsory secondary education	29.6	479
2nd compulsory secondary education	28.1	455
3rd compulsory secondary education	22.2	359
4th compulsory secondary education	12.3	199
Classes at A level	7.7	125
*Age*
12	16.2	262
13	26.2	424
14	22.1	358
15	17.0	275
16	12.1	195
17	6.4	103
*Nationality*		
Spanish	83.9	1357
Other European countries	8.2	133
American	4.8	77
African	2.4	38
Asian	0.6	10
Australian/Oceanian	0.1	2


### Correlations

As seen in Table 2, the Pearson’s bivariate correlations for the studied variables showed that victimization was positively correlated with depressive symptoms and suicidal thoughts and behaviors, and negatively correlated with gratitude. Gratitude was negatively correlated with depressive symptoms and suicidal ideation and behaviors.

**Table 2:** Descriptive statistics and bivariate correlations between the studied variables among total sample.

	Correlations					
Variable	M *(SD)*	Range	1	2	3	4
(1) Victimization	0.80 (0.73)	[0–4]				
(2) Depressive symptoms	1.51 (0.35)	[1–8]	0.38^∗∗∗^			
(3) Suicidal thoughts and behaviors	5.53 (3.87)	[3–22]	0.41^∗∗∗^	0.58^∗∗∗^		
(4) Gratitude	5.51 (1.12)	[1–7]	-0.26^∗∗∗^	-0.49^∗∗∗^	-0.41^∗∗∗^	


*^∗∗∗^p < 0.001*.

### Differences Between Girls’ and Boys’ Scores

Regarding gender differences, means in the variables were compared between girls and boys with Student’s *t*-test and with Cohen’s *d* to calculate the strength of the relationships (effect size). As seen in Table 3, girls scored higher than boys on depressive symptoms and suicidal thoughts and behaviors (*p* < 0.001; *d* = -0.35, and -0.37, respectively). No differences were found between girls and boys in victimization or on gratitude scores.

**Table 3:** Gender differences in the studied variables.

	Gender differences			
Variable	Male *M (SD)*	Female *M (SD)*	*T*	*d*
Victimization	0.78 (0.71) *n* = 800	0.82 (0.75) *n* = 817	-1.05	-0.05
Depressive symptoms	1.45 (0.31) *n* = 799	1.57 (0.37) *n* = 816	-7.22***	-0.35
Suicidal thoughts and behaviors	4.81 (3.02) *n* = 797	6.23 (4.44) *n* = 815	-7.53***	-0.37
Gratitude	5.52 (1.04) *n* = 798	5.50 (1.18) *n* = 816	0.36	0.02


*^∗∗∗^*p* < 0.001.*

### Moderating Effect of Gratitude

To test the moderation hypothesis for boys and girls, the moderating effect of gratitude in the relationship between victimization and suicide risk (both depressive symptoms and suicidal behavior and ideation) was estimated, using the PROCESS macro (Model 1) by Hayes (2013). The specifications of each model are summarized in Table 4.

**Table 4:** Moderating effect of gratitude on suicide risk for the female and male samples.

	Male sample					Female sample				
	b	SE	R^2^	Δ R^2^	95% CI	b	SE	R^2^	Δ R^2^	95% CI
**Depressive symptoms**			0.49^∗∗∗^					0.64^∗∗∗^		
Constant	1.11***	0.13			0.84–1.37	1.50***	0.16			1.19–1.81
Age	0.03*	0.01			0.01–0.05	-0.01	0.01			-0.03–0.02
School grade	-0.02	0.01			-0.04–0.01	0.05**	0.02			0.02–0.09
Victimization	0.09***	0.01			0.07–0.12	0.14***	0.02			0.11–0.17
Gratitude	-0.12***	0.01			-0.13–-0.09	-0.14***	0.01			-0.15–-0.12
Victimization × Gratitude	-0.02	0.01		0.00	-0.04–0.01	-0.03**	0.01		0.01^∗∗^	-0.05–-0.01
**Suicidal thoughts and behaviors**			0.42^∗∗∗^					0.60^∗∗∗^		
Constant	2.22	1.38			-0.49–4.93	3.43	1.98			-0.45–7.31
Age	0.21	0.12			-0.02–0.44	0.20	0.17			-0.14–0.53
School grade	-0.16	0.14			-0.43–0.11	-0.02	0.20			-0.41–0.37
Victimization	1.09***	0.14			0.80–1.37	2.06***	0.18			1.70–2.41
Gratitude	-0.71***	0.10			-0.90–-0.52	-1.33***	0.11			-1.55–-1.11
Victimization × Gratitude	-0.16	0.13		0.00	-0.40–0.09	-0.40**	0.13		0.01^∗∗^	-0.65–-0.15


*N_*males*_ = 798 (for deppresive symptoms) and 796 (for suicide thoughts and behaviors) N_*females*_ = 815; ^∗^*p* < 0.05, ^∗∗^*p* < 0.01, ^∗∗∗^*p* < 0.001.*

**FIGURE 1:**
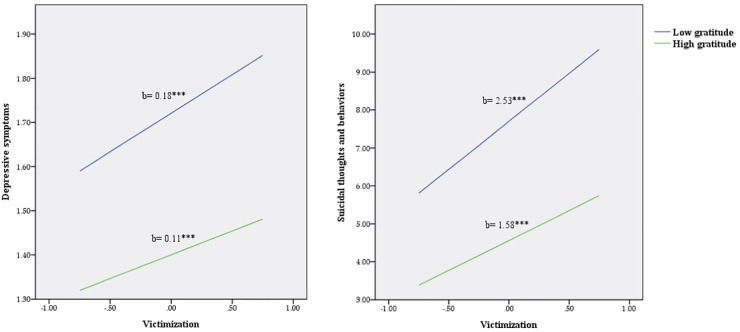
Relationship of victimization and gratitude for predicting suicide risk (depressive symptoms and suicidal thoughts and behaviors) in girls. ^∗∗∗^*p* < 0.001.

There was a significant effect of gratitude on depressive symptoms for both boys (*b* = -0.12, *p* < 0.001) and girls (*b* = -0.14, *p* < 0.001), as well as a significant association between gratitude and suicidal thoughts and behaviors for boys (*b* = -0.71, *p* < 0.001) and girls (*b* = -1.33, *p* < 0.001). However, there was only a significant interaction between victimization and gratitude scores on both depressive symptoms (b = -0.03, 95% CI: -0.15 to -0.12, *p* < 0.01) and suicidal thoughts and behaviors (*b* = -0.40, 95% CI: -0.65 to -0.15, *p* < 0.01) for girls. To visually inspect the interaction effects, two simple slope analyses were conducted (see Figure 1). This approach selects two arbitrary points (i.e., one standard deviation above and below the mean) of the moderator to estimate the effect of the predictor on the outcome. As seen in Figure 1, simple slope analysis revealed that among girls the positive association between victimization and depressive symptoms was weaker at high levels of gratitude (*b* = 0.11; 95% CI: 0.07, 0.15, *t* = 5.01, *p* < 0.001) compared to low levels (*b* = 0.18; 95% CI: 0.14, 0.21, *t* = 10.83, *p* < 0.001). In addition, Figure 1 shows that for girls, the positive relationship between victimization and suicidal thoughts and behaviors was weaker at high (*b* = 1.58; 95% CI: 1.06, 2.10, *t* = 5.94, *p* < 0.001) compared to low (*b* = 2.53; 95% CI: 2.14, 2.93, *t* = 12.63, *p* < 0.001) levels of gratitude.

## Discussion

This study aimed to examine the relationship between victimization and suicide risk in adolescents aged 12–17 years and to delve into the role played by gratitude in the bullying victimization-suicide risk relationship. Regarding the first hypothesis (H1), the results are in line with previous literature suggesting that adolescents who are bullied at school are more likely to show a decreased psychological adjustment and higher levels of depression and suicidal thoughts and behaviors(Hertz et al., 2013). In addition, and consistent with previous research (Wood et al., 2008; Wu et al., 2018), gratitude was negatively associated with victimization and positively associated with depressive symptoms and suicide.

Concerning the second hypothesis (H2), the results were in accordance with previous literature that found gender differences for psychological maladjustment in favor of female after suffering from bullying (Fredrick and Demaray, 2018). Specifically, girls reported higher depressive symptoms and suicidal behavior and ideation than their male counterparts. Further, the present findings support those of Bannink et al. (2014), who also found a relationship between gender and mental health problems and suicidal ideation in the bullying victimization context, even after controlling for baseline mental health and baseline suicidal ideation. Similarly, Fredrick and Demaray (2018) found that girls reported higher levels of depressive symptoms and suicidal ideation than boys, which tends to support the current findings.

Regarding bullying victimization, in line with previous research (Kowalski et al., 2014), our study found no gender differences. Thus, inconsistent results remain with respect to the role of gender in this area of concern (cf. Zych et al., 2015). Further, contrary to our expectations, no gender differences were found in levels of gratitude. While some researchers found gender differences in expression of gratitude (Petrocchi and Couyoumdjian, 2016; Disabato et al., 2017), other studies (Freitas et al., 2011; Ruch et al., 2014; Wang et al., 2015) are in line with our results. One plausible reason for this finding is that gratitude could be related to the age and maturation of participants. The majority of the adolescents we studied were aged between 12 and 14 years; it could be that, among these early adolescents, more complex forms of gratitude have yet to be developed (Merçon-Vargas et al., 2018). Another possible reason could be the influence of culture on gratitude development in children and adolescents. Indeed, some researchers (Tudge et al., 2016; Mendonça et al., 2018) have found differences across societies in the extent to which various types of gratitude were expressed in the age-related patterns of gratitude expression. Thus, both maturity and environmental conditions could influence the development of gender differences in the expression and experience of more complex forms of gratitude (Kashdan et al., 2009). Although some studies in other European countries (e.g., Germany; Ruch et al., 2014) also revealed no gender differences in adolescents, further research examining these potential differences in the Spanish culture are needed.

The last hypothesis (H3) was partially supported, as the moderation analyses results in the present study show that gratitude was associated with less suicide risk in girls, but not in boys. Although gratitude was linked to less suicide risk (emerging as a significant and negative predictor for both depressive symptoms and suicidal ideation and behaviors for girls and boys), when we tested the moderator role, it was solely significant for girls involved in victimization situations (both high- and low-victimization). That is, gratitude buffered the relationship between victimization and suicide risk, as high gratitude was related to lower levels of depression and suicidal ideation and behaviors, even in cases of high bullying victimization – but only for females. These results are consistent with earlier studies (Kashdan et al., 2009) finding more benefits of gratitude for females. A tentative explanation for this effect could be differences between girls and boys in the experience and management of negative emotions (Nolen-Hoeksema, 2012; Kim et al., 2018). Adolescent females have been found to be more susceptible to interpersonal stress (Hammen, 2009; Hankin et al., 2015) and to “experience more intense and prolonged tension as a result of interpersonal stress compared to males” (Kim et al., 2018, pp. 663). This suggests that girls may be at greater risk for depressive symptoms and suicide after experiencing relational aggression such as bullying victimization (Nolen-Hoeksema and Girgus, 1994; Bor et al., 2014). Further, whereas males are more likely to engage in reward-seeking and impulsive acts in response to negative emotions, females tend to be more conscious of and focused on their emotions and more likely to engage in ruminative thoughts when facing negative emotions associated with relational aggression experiences (Nolen-Hoeksema and Girgus, 1994; Nolen-Hoeksema, 2012). For some females, this awareness of and engagement with feelings may become maladaptive, in the form of a ruminative attention to emotions (Zahn-Waxler et al., 2008; Nolen-Hoeksema, 2012). Considering gratitude as the tendency to “possess a worldview that is more focused on the appreciation of the good things in life, including personal qualities, skills, and resources which may lead to less self-criticism when facing life circumstances” (Petrocchi and Couyoumdjian, 2016, pp. 200–201), women with higher gratitude may engage in a more positive reappraisal against negative emotions and rumination associated with stressful situations such as bullying victimization (Nezlek et al., 2018). According to Sergeant and Mongrain (2011), self-critical individuals are particularly responsive to the benefits of gratitude intervention. In this sense, as dispositional gratitude has been associated with less self-criticism and self-attacks (Petrocchi and Couyoumdjian, 2016), women could benefit from gratitude as a type of self-protective mechanism from thoughts about unwanted negative emotional experiences or adverse social consequences. However, this is a tentative explanation; future research is needed to better understand associated factors for these gender differences in the associations among victimization, gratitude, and mental health indicators.

Findings of the current research suggest that gratitude is a relevant protective factor for the prevention of suicide risk in victims of bullying, but only for girls. Although boys and girls show similar levels of gratitude, on the basis of the present findings, one could argue that gratitude is more important for girls in terms of preventing suicide risk in the context of bullying.

Following Fredrickson (1998, 2001) broaden-and-build theory, gratitude may contribute to individuals’ positive emotions, thereby broadening their momentary thought–action repertoires undoing the effects of negative emotions after a bullying experience and, besides, enduring personal resources that people use to regulate their experiences of negative emotions. Therefore, fostering gratitude not only among victims of bullying but also among adolescents may be good because of its effects on adolescents’ positive mood as well as constitute a way for achieving flourishing and a healthier life (Wood et al., 2010). When targeting efforts at promoting gratitude among adolescents, the different facets of this resource stated by McCullough et al. (2002) should be specifically addressed. In short, these authors highlight the relevance of the intensity experienced by a person after a positive event (intensity), the number of times experiencing gratitude each day (frequency), the number of life circumstances for which a person feels grateful at a given time (span) and the number of individuals to whom one feels grateful for a single positive outcome (density of gratitude). Finally, cultivating gratitude and its facets among adolescents would plausibly promote higher positive emotions and related subjective well-being, as well as lower negative emotions, depressive symptoms, and suicide risk.

### Theoretical and Practical Implications

There are several potential implications of the present research. Theoretically, the findings suggest that gratitude may protect individuals from stress and enable them to achieve more resilience, thus providing evidence for the utility of gratitude for promoting mental health (cf. Bono and Sender, 2018). Considering our findings, victims of bullying with higher gratitude could engage in more positive reappraisal strategies against negative acts such as bullying and, therefore, they may benefit from less self-criticism and self-blaming comparing to those who show less gratitude. However, future research should compare these effects of gratitude while understanding for the emotional impact and the psychological symptoms displayed by the victim. Thus, it is necessary to test whether high gratitude profiles would remain the same or alter within the same individual depending on the perceived severity of bullying or the length of time they are involved in these aggressive behaviors. In addition, our results showed that gratitude played a role as a moderator with this effects being conditioned by gender. It draws attention to the fact that gender development in adolescence involves the development of several emotional components that might affect the victim’s expression of gratitude. Therefore, the present research highlight the importance of examining hypotheses about explanations from theories on gender development involving expression and experience of gratitude to better understand these relations.

Practically, the present study could be quite informative for school staff and mental health professionals who deal with adolescent mental health consequences after bullying incidents. Clinicians, school personnel and school policymakers should take a step toward taking more notice of the development of protective factors against bullying victimization that might reduce its pernicious effects and lead to more positive outcomes (Hemphill et al., 2014). In so doing, we would suggest addressing both well-being and mental health difficulties through prevention and early intervention and providing safer contexts in which adolescents with mental health difficulties can be supported (cf. Hymel et al., 2018). However, the role of gender should be considered in developing optimal preventive interventions and service. The results of the present study support efforts to teach gratitude to girls who are victims of bullying and may be vulnerable to depressive symptoms and suicidal thoughts and behaviors. This implies that, among girls, thankfulness exercises could also be integrated into modules for mitigating the consequences of experiencing bullying and reducing their consequent emotional and behavioral difficulties. Considering that anti-bullying programs are more effective when targeting vulnerable adolescents (Bradshaw, 2015), these findings support calls for developing approaches that are sensitive to gender differences on the impact of bullying in emotional and behavioral outcomes.

### Limitations and Strengths

Findings of this research should be interpreted within the context of several limitations, including the cross-sectional nature, self-reported measures, and a convenience sampling method. First, the cross-sectional design of the study prevents us from establishing causal relationships among the variables. Future research could include longitudinal assessments in order to examine the directionality and combined effect among gratitude, depressive symptoms, gender and suicide thoughts and behaviors in victimized adolescents. Second, this study used self-report scales to assess victimization, gratitude, and suicide risk. Although these instruments were selected for their good psychometric properties, it is possible that the nature of the self-report measures could be lend itself to bias (i.e., social desirability). Future studies with a multi-method measurement approach (e.g., peer nomination method for bullying victimization or clinical assessment for depressive symptoms and suicidal ideation) are needed to generalize the results of this study. Researchers could also examine whether gratitude plays a buffering role in the consequences of other types of bullying victimization (e.g., cybervictimization). Finally, the convenience sample limits the extent to which these findings can be generalized. Future studies should use more representative samples and could, for instance, compare clinical and non-clinical samples.

Despite these limitations, this research adds to the gratitude literature in many ways. We note that the explorative analyses used allowed us to examine the relationship between bullying victimization, gratitude and suicide risk, and to better understand the role of gratitude in this complex relationship. As far as we know, it is the first known study to explore the buffering role of gratitude in the relationship between bullying victimization and suicide risk in adolescence. Another strong point refers to focusing on the identification of protective individual factors that allowed us to identify gratitude as a relevant factor that should be included in programs aimed at the prevention and treatment of suicidality and, similarly, in programs to help adolescents cope with stress and the negative consequences of bullying experiences.

## Conclusion and Directions for Future Research

The present research sheds light on the associations between bullying victimization, gratitude and suicide risk in adolescent boys and girls, and, besides, contributes to the scarce literature on the moderating role of gratitude. The findings emphasize the relevance of gender differences analyses when investigating depression and suicide, and highlight the importance of gender-tailored development and evaluation of intervention studies. The results suggest that boys and girls may not benefit in the same manner from gratitude after experiencing face-to-face bullying. Future development of positive psychology and bullying research and interventions should take this into account.

## Data Availability

All datasets generated for this study are included in the manuscript and/or the supplementary files.

## Author Contributions

All authors participated and contributed in the conception of the study, in performing the data collection and statistical analysis, and in writing the manuscript. The final manuscript has been approved by all authors.

## Conflict of Interest Statement

The authors declare that the research was conducted in the absence of any commercial or financial relationships that could be construed as a potential conflict of interest.
